# Quantitative Fit Test of a 3D Printed Frame Fitted Over a Surgical Mask: An Alternative Option to N95 Respirator

**DOI:** 10.1155/2022/1270106

**Published:** 2022-03-16

**Authors:** Suchada Kongkiatkamon, Norachai Wongkornchaowalit, Valailuck Kiatthanakorn, Somkiat Tonphu, Chaiyos Kunanusont

**Affiliations:** ^1^Bangkok Hospital Dental Center, Bangkok Hospital, Bangkok 10310, Thailand; ^2^Bangkok Health Research Center (BHRC), Bangkok Dusit Medical Services Public Company Limited (BDMS), Bangkok, Thailand

## Abstract

**Background:**

COVID-19 has spread worldwide and caused severe acute respiratory syndrome coronavirus 2 (SARS-CoV-2) led to numerous dead cases. However, with the new COVID-19 outbreaks, there is a shortage of personal protective equipment (PPE) especially N95 masks worldwide including Thailand. This issue had placed the health professional in great need of an alternative mask.

**Aim:**

This study aimed to measure the fit factor of 3D printed frames by quantitative fit testing (QNFT) to find an alternative facemask by using a mask fitter together with 2 different kinds of the American Society for Testing and Materials (ASTM) level 1 surgical mask.

**Materials and Methods:**

Two commonly used surgical masks (Sultan Com-Fit Super Sensitive Ear Loop Mask or “White Mask Group,” not water-resistant, and Sultan Blue Com-Fit Super High Filtration Ear Loop Mask or “Blue Mask Group,” water-resistant) with and without 3D printed frame covering. The fit performance was measured by a quantitative fit test (QNFT) device (PortaCount, model 8048, TSI Incorporated, Minnesota, USA) accepted by the Occupational Safety and Health Administration (OSHA). The PortaCount device, which is based on a miniature continuous flow condensation nucleus counter (CNC), assesses the respiratory fit by comparing the concentration of ambient dust particles outside the surgical mask to the concentration that has leaked into the surgical mask. The ratio of these two concentrations (C_out_/C_in_) is called the fit factor. A fit factor of a 3D printed frame of at least 100 is required and considered as a pass level.

**Results:**

We found that the mask fitter improves the overall performance of surgical masks significantly. The improved performance is comparable to that of N95.

**Conclusion:**

The mask fitter improves the performance of surgical masks. The authors suggested that further study on frame material, shape, and expanded sample size would be beneficial to society.

## 1. Introduction

COVID-19 is considered a pandemic disease in this era and rapidly spread worldwide, causing severe acute respiratory syndrome coronavirus 2 (SARS-CoV-2) and numerous dead cases [1, 2]. The spread of severe acute respiratory syndrome coronavirus 2 (SARS-CoV-2) occurs mainly via respiratory droplets [3, 4]. During dental treatment procedures, many droplets and aerosols are generated, and the standard protective measures are not effective enough to prevent the spread of COVID-19 in dental clinics and hospitals [3, 5, 6].

One of the essential personal protective equipment (PPE) for healthcare practitioners is respiratory protective equipment (RPE) [7]. The standard surgical face mask is one of the RPE designed to protect the nasal and oral mucosa from splashes and droplets. Even though the filter efficiency test is carried out and evaluated by the manufacturers according to the FDA regulations, the fit performance plays a great role that we should not ignore.

Because the surgical mask fits loosely to the wearer's face, perimeter leakage during the procedure may carry aerosol particles from dental aerosol-generating procedures. A total of 10% to 40% of particles penetrate the facial seal as a result of poor fit [8]. Since it is found that the coronavirus can survive in the air for hours, the World Health Organization (WHO) and the United States Centers for Disease Control and Prevention (US CDC) recommended a new “airborne precaution” for medical professionals [7]. Thus, only filtration performance is not enough to protect the clinical practitioners from airborne substances, and a respiratory protective device should have high filtration efficiency with sufficient fit as well.

However, with the new COVID-19 outbreaks, there is a shortage of PPE especially N95 masks worldwide including Thailand. This issue had placed the health professional in great need of an alternative mask. The research also shows that the effectiveness of N95 is sometimes not different from that of a surgical mask [9].

Recently, the Bellus3D team has been collaborating with the researcher at Loma Linda University School of Dentistry to develop a mask fitter by simply performing a 3D facial scan by using an application such as Bellus3D Face App and Bellus3D Dental Pro [10]. From the facial scan, a customized mask fitter is designed and can be printed easily from a 3D printer. The covering of a customized mask fitter is designed to improve the peripheral seal of the surgical mask. The fit performance was qualitatively tested by comparing the American Society for Testing and Materials (ASTM) level 1, 2, and 3 surgical masks to N95 ASTM F2100-11 (2011) [11]. They found that ASTM level 2 and 3 masks with the personalized frame had a better seal and prevented the aromatics from being detected inside the mask.

This study aimed to test the fit factor of a 3D printed frame by quantitative fit testing (QNFT) to find an alternative facemask by using a mask fitter together with 2 different kinds of ASTM level 1 surgical masks.

## 2. Materials and Methods

**Figure 1:**
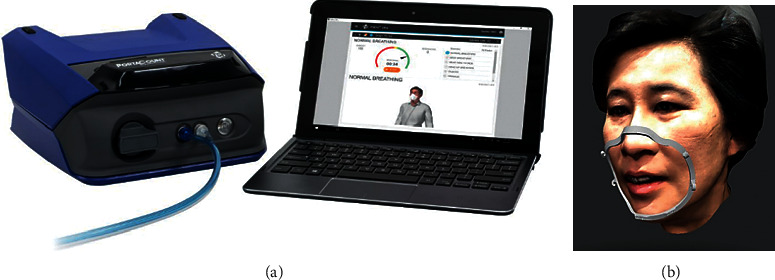
PortaCount and Face Frame. (a) PortaCount respirator fit tester, (b) 3D facial scan, and designed mask fitter from Bellus3D Face App and Bellus3D Dental Pro.

Five dentists ranging in age from 37 to 55 years were recruited in this study to test the two commonly used surgical masks (Sultan Com-Fit Super Sensitive Ear Loop Mask or “White Mask Group,” not water-resistant and Sultan Blue Com-Fit Super High Filtration Ear Loop Mask or “Blue Mask Group,” water-resistant) with and without 3D printed frame covering. The fit performance was measured by a quantitative fit test (QNFT) device (PortaCount, model 8048, TSI Incorporated, Minnesota, USA) accepted by the Occupational Safety and Health Administration (OSHA) [12]. The PortaCount device (Figure 1), which is based on a miniature continuous flow condensation nucleus counter (CNC), assesses the respiratory fit by comparing the concentration of ambient dust particles outside the surgical mask to the concentration that has leaked into the surgical mask [13]. The ratio of these two concentrations (C_out_/C_in_) is called the fit factor [8, 13]. A fit factor of at least 100 is required and is considered as a pass level. The 3D facial scan and designed mask fitter from Bellus3D Face App and Bellus3D Dental Pro. Similarly, we used polylactic acid (PLA) following the Bellus3D recommendation.

The adaptor is attached to one side of the surgical mask then connect the tube from PortaCount to the adaptor port. Subjects performed 2 minutes and 29 seconds of exercises in the bending over position, talking, head side-to-side position, and head up-and-down position (Table 1) in a 25°C air-conditioned room. The QNFT was performed in three groups: blue mask, blue mask with a frame, and white mask with a frame.

## 3. Results

**Figure 2:**
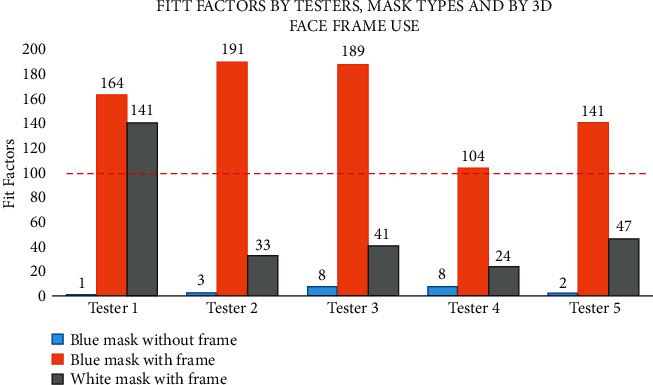
Summarized quantitative fit test results with interpretation. Describe “Head Tilt” results specifically for dentists—(5/5 tests) passed.

Although the five testers were different in terms of age and gender, we found a consistent improvement in terms of fit factors when face frames were applied. The type of mask also played an important role in passing the 100 fit factor threshold (Figure 2 and Table 2). Only when a blue mask (water-resistant) was used with a face frame, fit factors were above the 100 thresholds. On the contrary, when either (a) a blue mask (water-resistant) without a face frame was tested, or (b) a white mask (not water-resistant) was used with a face frame, fit factors were below 100.

## 4. Discussion

Medical and dental practices are severely affected by COVID-19 [14, 15]. Due to the shortages or limited PPE especially N95, leaving doctors, nurses, and other frontline workers dangerously ill-equipped to care for COVID-19 patients, a reusable PPE-like facemask was recommended. In addition, people are psychologically affected from COVID-19 [16, 17].

There has also been discussion about the reuse of N95 respirators after sterilization with ionizing radiation, UV, or heat. Following sterilization, it can cause in decline in their filtering efficiency due to damage to the respirators [18]. Disposable N95 masks pass the qualitative fit-test but have decreased filtration efficiency after cobalt-60 gamma irradiation [19]. Ideally, healthcare workers in true need of N95 respirators should be using them as they are designed and disposing of them when appropriate.

Quantitative fit tests are considered valid measures and normally tested in tight-fitting respirators; however, the same principle is applied to measure the fit performance of surgical masks and surgical masks covered with mask fitter in this study [20, 21]. Even if the mask fitter tightens the surgical mask, the user-seal-check may be unreliable for detecting leakage. In some cases, users reported that the respirator fitted well, but the fit factor was very low, and the overall quantitative fit test failed [22]. Thus, the leakage between the face and the respirator is not easily detected by the user. The QNFT is the gold standard used to determine this fit objectively. In this study, the authors tested the fit factor by QNFT PortaCount to find an alternative facemask by using a mask fitter together with 2 different kinds of ASTM level 1 surgical masks.

In this study, the customized 3D printed mask fitter improves the quantitative fit performance of the surgical mask. But differences can be seen between groups 1 and 2. Group 1 (Sultan Com-Fit Super Sensitive Ear Loop Mask or “White Mask Group”) is the ASTM mask level 1 in white color which the outer facing is made of polypropylene and an aluminum strip was incorporated at the nose piece. However, group 2 (Sultan Blue Com-Fit Super High Filtration Ear Loop Mask or “Blue Mask Group”) is the ASTM mask level 1 in blue color which the outer facing is a double layer (fractured film and cellulose) and aluminum with synthetic foam support was incorporated at the nose piece. The authors believe that nasal support played a great role in the fitting [23]. From this study, head tilt, which is the usual position of dental practice, did not compromise performance. However, talking showed the most compromised results. It was worth pointing out that the fit factor of Tester 4 was lower than the other testers. We had taken note that, during the talking position, Tester 4 talked at a fast speed. This might have contributed to the accumulation of particles inside (Cin), thus decreasing the overall fit factor.

Recently, a study by Liu et al. [24] mentioned that 90% of subjects passed the minimum requirement of QNFT by using mask fitters over the ASTM mask level 3 which has higher bacterial filtration efficiency (BEF) than the ASTM level 1. However, in our study, we used mask fitter over ASTM level 1 which provides a good fit and comparable result to N95. So, it is possible that the design of the mask, cellulose lining material, or sponge antifog nose bridge pad would have a high potential effect on the fit than BFF properties.

Similarly, we used polylactic acid following the Bellus3D recommendation. However, the other materials that could be used should be environmentally friendly, biodegradable, flexible physical property, and economical. Elastic tightening can be done to hold the fitter in place around the head [25]. Although Bella3D recommends using a chain of thin rubber bands, it can be possible to use elastic cloth or string.

Some other alternatives to disposable N95 respirators can be reusable stop-gap respirators as alternatives made from 3D printing, silicone molding, and plastic extrusion [26–28]. Anwari et al. [26] developed and did the preliminary testing of an open-hardware-licensed device, the “simple silicone mask” (SSM). The respirator originally included a cartridge for holding filter material; this was modified to connect to standard heat-moisture exchange (HME) filters (N95 or greater) after the cartridge showed poor filtration performance due to flow acceleration around the filter edges, which was exacerbated by high filter resistance. All 8 HME-based iterations provided an adequate seal by user seal checks and achieved a pass rate of 87.5% (*N* = 8) on quantitative testing, with all failures occurring in the first iteration. The overall median fit-factor was 1662 (100 = pass). The estimated unit cost for a production run of 1000 using distributed manufacturing techniques is CAD $15 in materials and 20 minutes of labor. Small-scale manufacturing of an effective, reusable N95 respirator during a pandemic is feasible and cost-effective. Required quantities of reusables are more predictable and less vulnerable to supply chain disruption than disposables. With further evaluation, such devices may be an alternative to disposable respirators during public health emergencies. The respirator described previously is an investigational device and requires further evaluation and regulatory requirements before clinical deployment. The authors and affiliates do not endorse the use of this device at present. Similarly, Ng et al. [25] developed one such device, the “SSM.” They evaluated the qualitative fit test (QNFT), comfort, breathability, and communication. The SSM scored 3.5/5 and 4/5 for comfort and breathability. The median overall harmonic mean fit factors of disposable N95 and SSM were 137.9 and 6316.7, respectively. SSM scored significantly higher than disposable respirators in fit-test runs and overall fit-factors (*p* < 0.0001). Overall passing rates in disposable and SSM respirators on QNFT were 65% and 100%. During dynamic runs, passing rates in disposable and SSM respirators were 68.1% and 99.4%, respectively; harmonic means were 73.7 and 1643. They validated the reusable N95 stop-gap filtering face piece respirator that can match existent commercial respirators which can be used in an emergency.

The mask fitter designed in this study is customized and has a better fit, but the N95 is not customized. But the N95 has its importance as it offers good prevention. In addition, the mask fitter is reusable. The mask filters themselves are not reusable to the same degree, but their use can be prolonged. To clinically evaluate such fitting devices, more clinical studies are needed. Aesthetic and pragmatic human performance considerations are equally important including comfort and breathability which were not carried out in our study. Further study on frame material, shape, and expanded sample size would be beneficial to society. This study can be expanded to include these factors in more sample sizes in a larger population.

## 5. Conclusion

From this study, we found that the 3D printed frame fitted over a surgical mask offers advantages comparable to those offered by N95 respirators. However, the fit and seal of the mask would be decreased once the speech pace increases. Also, the surgical mask brand or even design would affect the result too. So, the author suggested that minimizing conversation and slow-speed talking would be very beneficial.

The custom mask fitter requires further investigation to test its effectiveness through quantitative means and further design adjustments to improve its comfort, user-friendliness, and everyday feasibility. In its current state, it cannot replace the N95 respirator but may provide an alternative PPE solution when N95 supplies are limited.

## Figures and Tables

**Table 1:** Modified ambient aerosol CNC quantitative fit testing (PortaCount) protocol for filtering facepiece respirators [12].

Exercises	Exercise procedure	Measurement procedure	Duration (seconds)
Bending over	The test subject shall bend at the waist as if going to touch his/her toes for 50 sec and inhale 2 times at the bottom	A 20-sec ambient sample, followed by a 30-sec mask sample	50

Talking	The test subject shall talk out loud, slowly and loud enough to be heard clearly by the test conductor for 30 sec. He/she will either read from a prepared text such as the rainbow passage, count backward from 100, or recite a memorized poem or song	A 30-sec mask sample	30

Head side-to-side	The test subject shall stand in place, slowly turning his/her head from side-to-side for 30 sec and inhale 2 times at each extreme	A 30-sec mask sample	30

Head up-and-down	The test subject shall stand in place, slowly moving his/her head up and down for 39 sec and inhale 2 times at each extreme	A 30-sec mask sample followed by a 9-sec ambient sample	39

Total duration			2 min and 29 sec

**Table 2:** Quantitative fit factors by testers and types of masks and framing.

SN	Mask type	Tester	Fit factor
1	Blue	Female	1
2	Blue	Female	3
3	Blue	Male	8
4	Blue	Male	8
5	Blue	Female	2
6	Blue + frame	Female	164
7	Blue + frame	Female	191
8	Blue + frame	Male	189
9	Blue + frame	Male	109
10	Blue + frame	Female	250
11	White + frame	Female	141
12	White + frame	Female	33
13	White + frame	Male	41
14	White + frame	Male	24
15	White + frame	Male	47

## Data Availability

The data used to support the findings of this study are available from the corresponding author upon request.

## References

[1] Zhou F., Yu T., Du R. (2020). Clinical course and risk factors for mortality of adult inpatients with COVID-19 in Wuhan, China: a retrospective cohort study. *The Lancet*.

[2] Zu Z. Y., Jiang M. D., Xu P. P. (2020). Coronavirus disease 2019 (COVID-19): a perspective from China. *Radiology*.

[3] Yu J., Zhang T., Zhao D., Haapasalo M., Shen Y. (2020). Characteristics of endodontic emergencies during coronavirus disease 2019 outbreak in Wuhan. *Journal of Endodontics*.

[4] Wang D., Hu B., Hu C. (2020). Clinical characteristics of 138 hospitalized patients with 2019 novel coronavirus-infected pneumonia in Wuhan, China. *The Journal of the American Medical Association*.

[5] Meng L., Hua F., Bian Z. (2020). Coronavirus disease 2019 (COVID-19): emerging and future challenges for dental and oral medicine. *Journal of Dental Research*.

[6] Rokaya D. (2020). COVID-19: prosthodontic challenges and opportunities in dental practice. *Journal of Advanced Oral Research*.

[7] van Doremalen N., Bushmaker T., Morris D. H. (2020). Aerosol and surface stability of SARS-CoV-2 as compared with SARS-CoV-1. *New England Journal of Medicine*.

[8] Oberg T., Brosseau L. M. (2008). Surgical mask filter and fit performance. *American Journal of Infection Control*.

[9] Smith J. D., MacDougall C. C., Johnstone J., Copes R. A., Schwartz B., Garber G. E. (2016). Effectiveness of N95 respirators versus surgical masks in protecting health care workers from acute respiratory infection: a systematic review and meta-analysis. *Canadian Medical Association Journal*.

[10] Hackleman D. (2020). LLU School of Dentistry and Bellus3D collaborate on mask solutions during COVID-19. https://dentistry.llu.edu/about/school-news/llu-school-dentistry-and-bellus3d-collaborate-mask-solutions-during-covid-19.

[11] Forouzandeh P., O’Dowd K., Pillai S. C. (2021). Face masks and respirators in the fight against the COVID-19 pandemic: an overview of the standards and testing methods. *Safety Science*.

[12] Lam S. C., Lee J. K. L., Yau S. Y., Charm C. Y. C. (2011). Sensitivity and specificity of the user-seal-check in determining the fit of N95 respirators. *Journal of Hospital Infection*.

[13] Bellus3D Software (2021). How to make bellus3D’s face mask fitter. https://bellus3d.com/solutions/facemask.html.

[14] Humagain M., Humagain R., Rokaya D. (2020). Dental practice during COVID-19 in Nepal: a descriptive cross-sectional study. *Journal of the Nepal Medical Association*.

[15] Beshyah S. A., Ibrahim W. H., Hajjaji I. M., Mami F. B., Arekat M., Abdelmannam D. K. (2020). Impact of the COVID-19 pandemic on clinical practice, medical education, and research: an international survey. *Tunisie Medicale*.

[16] Passavanti M., Argentieri A., Barbieri D. M. (2021). The psychological impact of COVID-19 and restrictive measures in the world. *Journal of Affective Disorders*.

[17] Rokaya D., Koontongkaew S. (2020). Can coronavirus disease-19 lead to temporomandibular joint disease?. *Open Access Macedonian Journal of Medical Sciences*.

[18] Schumm M. A., Hadaya J. E., Mody N., Myers B. A., Maggard-Gibbons M. (2021). Filtering facepiece respirator (N95 respirator) reprocessing. *JAMA*.

[19] Cramer A., Tian E., Galanek M. (2020). Assessment of the qualitative fit test and quantitative single-pass filtration efficiency of disposable N95 masks following Gamma irradiation. *JAMA Network Open*.

[20] Han D.-H., Willeke K., Colton C. E. (1997). Quantitative fit testing techniques and regulations for tight-fitting respirators: current methods measuring aerosol or air leakage, and new developments. *American Industrial Hygiene Association Journal*.

[21] Zhuang Z., Bergman M., Lei Z., Niezgoda G., Shaffer R. (2017). Recommended test methods and pass/fail criteria for a respirator fit capability test of half-mask air-purifying respirators. *Journal of Occupational and Environmental Hygiene*.

[22] Karuppasamy K., Obuchowski N. (2021). Comparison of fit for sealed and loose-fitting surgical masks and N95 filtering facepiece respirators. *Annals of Work Exposures and Health*.

[23] Brill A. K., Pickersgill R., Moghal M., Morrell M. J., Simonds A. K. (2018). Mask pressure effects on the nasal bridge during short-term noninvasive ventilation. *ERJ Open Research*.

[24] Liu J., Ma J., Ahmed I. I., Varma D. K. (2021). Effectiveness of a 3D-printed mask fitter in an Ophthalmology setting during COVID-19. *Canadian Journal of Ophthalmology*.

[25] Ng W. C. K., Mbadjeu Hondjeu A. R., Syrett A. (2020). Subject validation of reusable N95 stop-gap filtering facepiece respirators in COVID-19 pandemic. *PLoS One*.

[26] Anwari V., Ng W. C. K., Mbadjeu Hondjeu A. R. (2021). Development, manufacturing, and preliminary validation of a reusable half-face respirator during the COVID-19 pandemic. *PloS One*.

[27] (2004). Fit testing procedures (mandatory). https://www.osha.gov/laws-regs/regulations/standardnumber/1910/1910.134AppA.

[28] Mueller A. V., Eden M. J., Oakes J. M., Bellini C., Fernandez L. A. (2020). Quantitative method for comparative assessment of particle removal efficiency of fabric masks as alternatives to standard surgical masks for PPE. *Matter*.

